# The genome sequence of the Brown Ash Ermine moth, *Zelleria hepariella *Stainton, 1849

**DOI:** 10.12688/wellcomeopenres.21495.1

**Published:** 2024-05-09

**Authors:** Douglas Boyes, Clare Boyes, University of Oxford and Wytham Woods Genome Acquisition Lab, Darwin Tree of Life Barcoding collective, Wellcome Sanger Institute Tree of Life Management, Samples and Laboratory team, Wellcome Sanger Institute Scientific Operations: Sequencing Operations, Wellcome Sanger Institute Tree of Life Core Informatics team, Tree of Life Core Informatics collective, Darwin Tree of Life Consortium

**Affiliations:** 1 UK Centre for Ecology & Hydrology, Wallingford, England, UK; 2 Independent researcher, Welshpool, Wales, UK

**Keywords:** Zelleria hepariella, Brown Ash Ermine moth, genome sequence, chromosomal, Lepidoptera

## Abstract

We present a genome assembly from a male *Zelleria hepariella* (the Brown Ash Ermine; Arthropoda; Insecta; Lepidoptera; Yponomeutidae). The genome sequence is 428.8 megabases in span. Most of the assembly is scaffolded into 19 chromosomal pseudomolecules, including the Z sex chromosome. The mitochondrial genome has also been assembled and is 16.31 kilobases in length. Gene annotation of this assembly on Ensembl identified 15,718 protein coding genes.

## Species taxonomy

Eukaryota; Opisthokonta; Metazoa; Eumetazoa; Bilateria; Protostomia; Ecdysozoa; Panarthropoda; Arthropoda; Mandibulata; Pancrustacea; Hexapoda; Insecta; Dicondylia; Pterygota; Neoptera; Endopterygota; Amphiesmenoptera; Lepidoptera; Glossata; Neolepidoptera; Heteroneura; Ditrysia; Yponomeutoidea; Yponomeutidae; Yponomeutinae; Zelleria; *Zelleria hepariella* Stainton, 1849 (NCBI:txid1594360).

## Background

*Zelleria hepariella,* Brown Ash Ermine is a micro-moth in the family Yponomeutidae. It is local throughout the UK, and is found throughout Europe ( GBIF Secretariat, 2024). The chestnut brown adult (forewing length 5–7.5 mm) has a distinctive head-down resting posture with the tip of the forewings slightly curved which results in a hook-tipped appearance ( Sterling *et al.*, 2012), making the adult of this species relatively easy to identify.

*Zellaria hepariella* lays its eggs on ash or occasionally on privet, and the larvae feed at the tips of branches in a dense spinning of leaf-tips. There are often several larvae in each web ( Emmet, 1996). The larvae pupate in July in a thick cocoon which is attached to a leaf of the host plant. The adult moth flies from July, spending the winter hibernating in dense vegetation such as yew or juniper, before emerging in the spring to mate ( Langmaid *et al.*, 2018). It comes to light.

The genome of *Zelleria hepariella* was sequenced as part of the Darwin Tree of Life Project, a collaborative effort to sequence all the named eukaryotic species in the Atlantic Archipelago of Britain and Ireland. Here we present a chromosomally complete genome sequence for *Zelleria hepariella* based on a male specimen from Wytham Woods, Oxfordshire, UK.

## Genome sequence report

**Figure 1.**
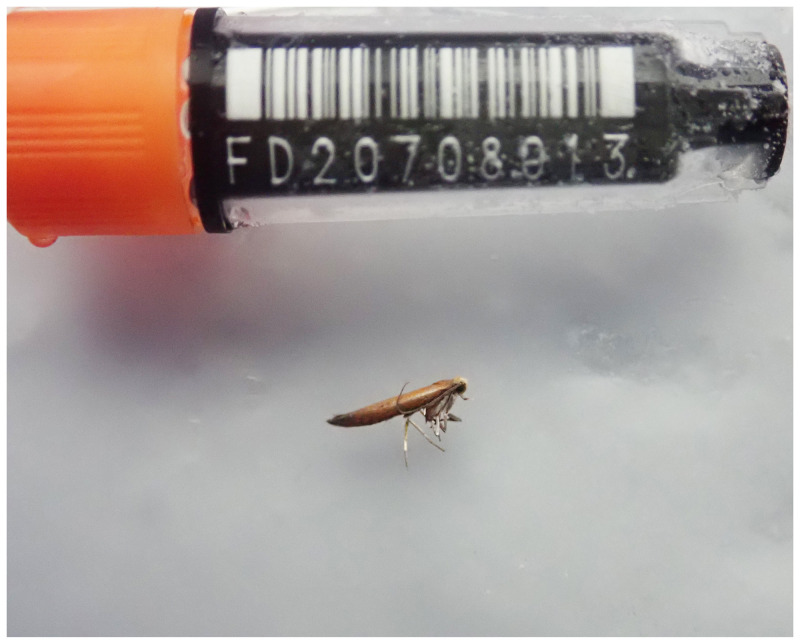
Photograph of the *Zelleria hepariella* (ilZelHepa1) specimen used for genome sequencing.

The genome was sequenced from a male *Zelleria hepariella* ( Figure 1) collected from Wytham Woods, Oxfordshire, UK (51.77, –1.34). A total of 35-fold coverage in Pacific Biosciences single-molecule HiFi long reads was generated. Primary assembly contigs were scaffolded with chromosome conformation Hi-C data. Manual assembly curation corrected 187 missing joins or mis-joins and removed 79 haplotypic duplications, reducing the assembly length by 2.69% and the scaffold number by 24.58%.

**Figure 2.**
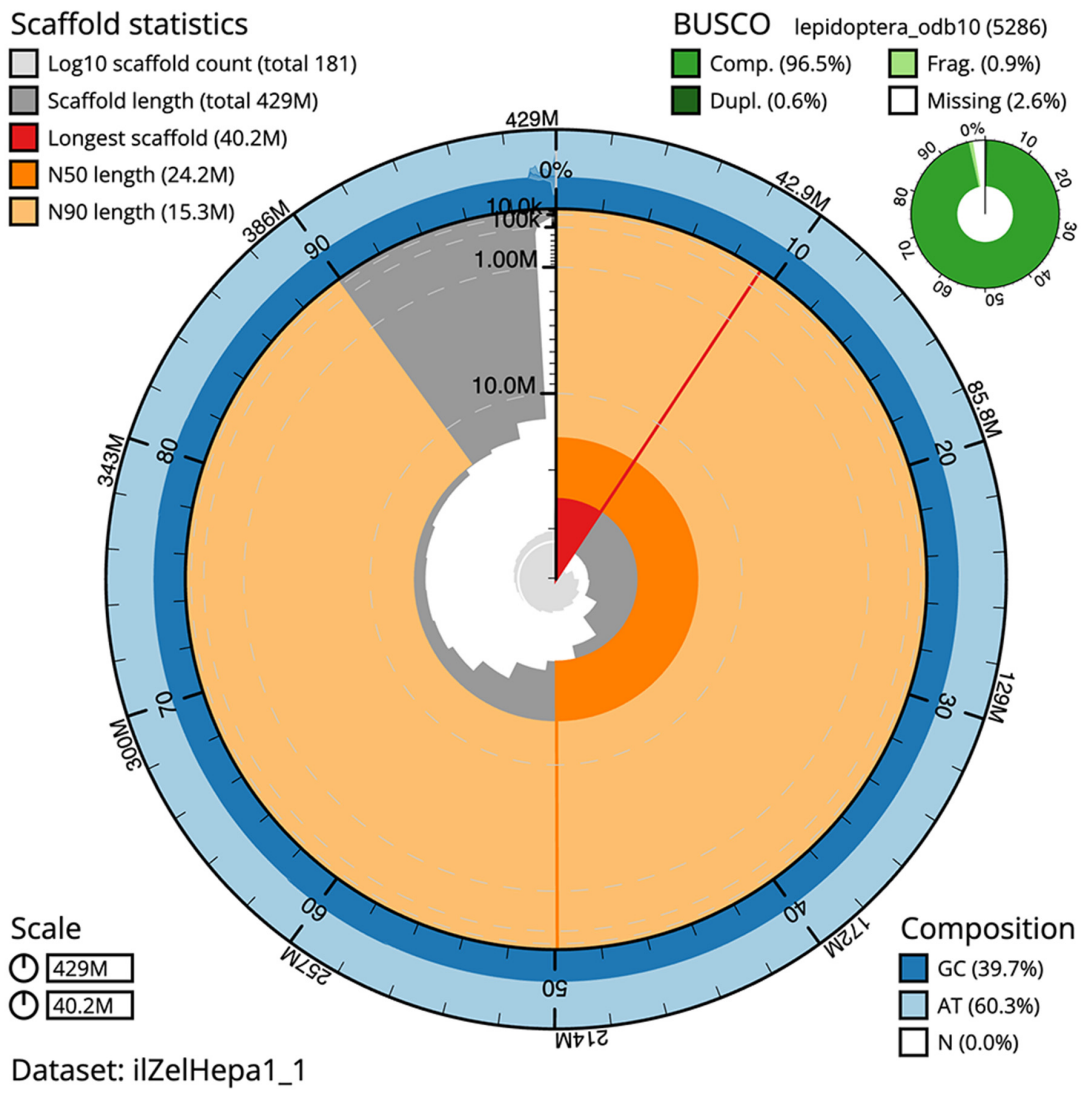
Genome assembly of *Zelleria hepariella*, ilZelHepa1.1: metrics. The BlobToolKit snail plot shows N50 metrics and BUSCO gene completeness. The main plot is divided into 1,000 size-ordered bins around the circumference with each bin representing 0.1% of the 428,786,765 bp assembly. The distribution of scaffold lengths is shown in dark grey with the plot radius scaled to the longest scaffold present in the assembly (40,238,452 bp, shown in red). Orange and pale-orange arcs show the N50 and N90 scaffold lengths (24,231,187 and 15,286,053 bp), respectively. The pale grey spiral shows the cumulative scaffold count on a log scale with white scale lines showing successive orders of magnitude. The blue and pale-blue area around the outside of the plot shows the distribution of GC, AT and N percentages in the same bins as the inner plot. A summary of complete, fragmented, duplicated and missing BUSCO genes in the lepidoptera_odb10 set is shown in the top right. An interactive version of this figure is available at https://blobtoolkit.genomehubs.org/view/ilZelHepa1_1/dataset/ilZelHepa1_1/snail.

**Figure 3.**
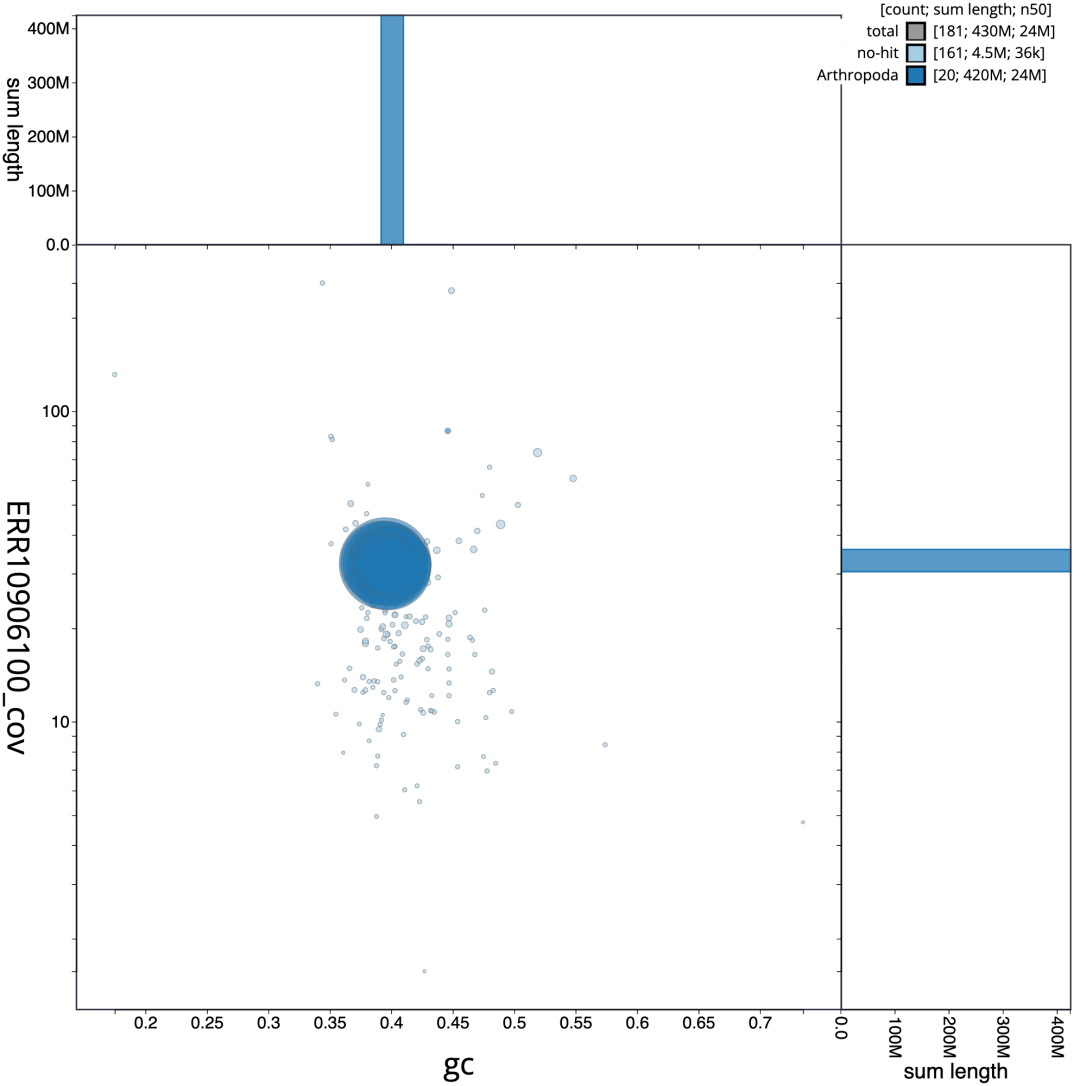
Genome assembly of *Zelleria hepariella*, ilZelHepa1.1: BlobToolKit GC-coverage plot. Sequences are coloured by phylum. Circles are sized in proportion to sequence length. Histograms show the distribution of sequence length sum along each axis. An interactive version of this figure is available at https://blobtoolkit.genomehubs.org/view/ilZelHepa1_1/dataset/ilZelHepa1_1/blob.

**Figure 4.**
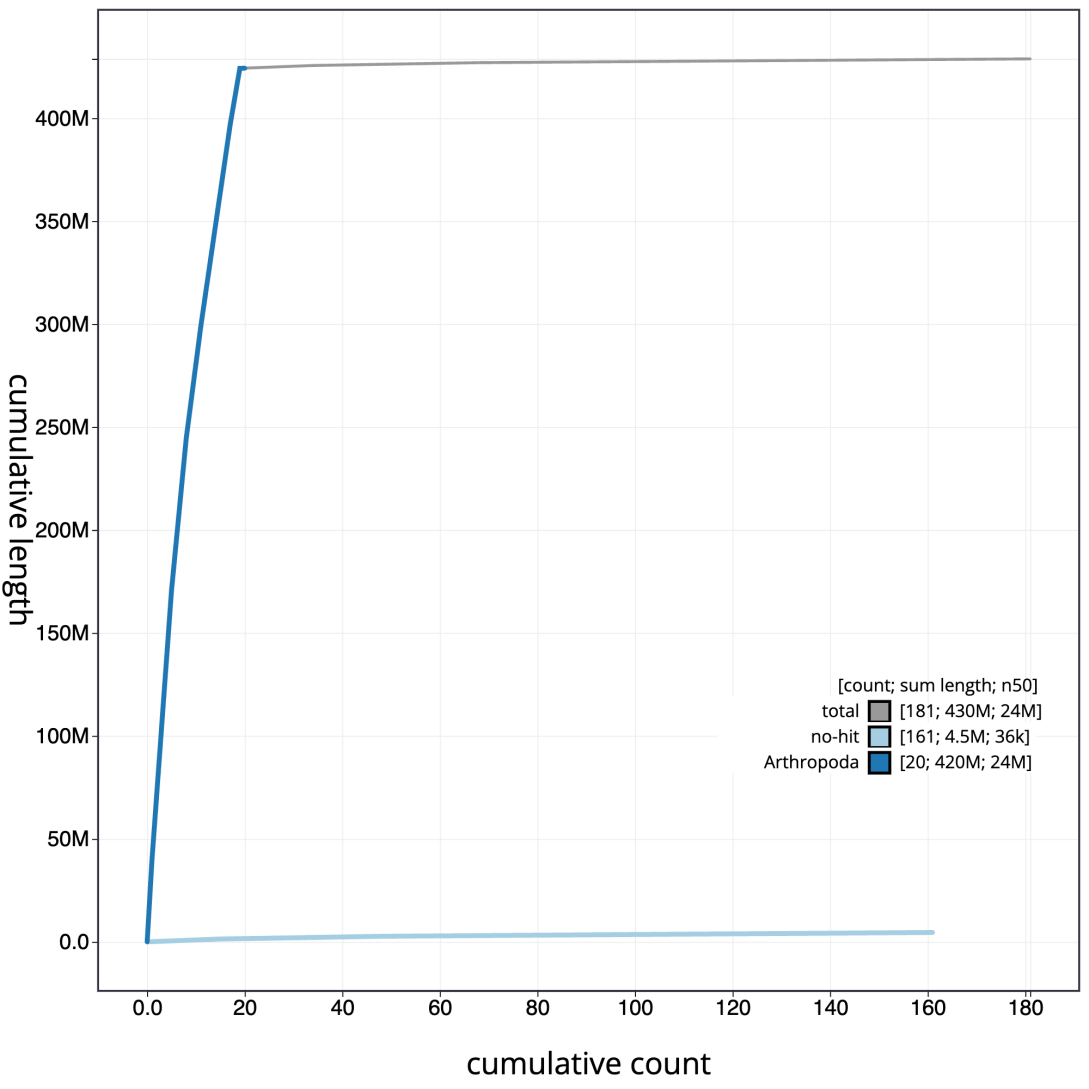
Genome assembly of *Zelleria hepariella*, ilZelHepa1.1: BlobToolKit cumulative sequence plot. The grey line shows cumulative length for all sequences. Coloured lines show cumulative lengths of sequences assigned to each phylum using the buscogenes taxrule. An interactive version of this figure is available at https://blobtoolkit.genomehubs.org/view/ilZelHepa1_1/dataset/ilZelHepa1_1/cumulative.

**Figure 5.**
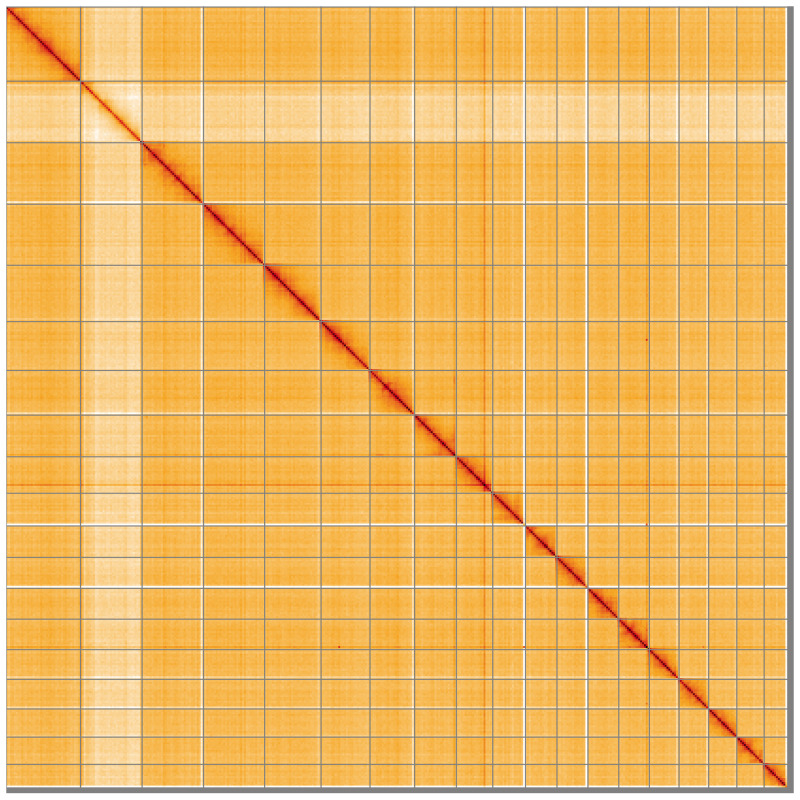
Genome assembly of *Zelleria hepariella*, ilZelHepa1.1: Hi-C contact map of the ilZelHepa1.1 assembly, visualised using HiGlass. Chromosomes are shown in order of size from left to right and top to bottom. An interactive version of this figure may be viewed at https://genome-note-higlass.tol.sanger.ac.uk/l/?d=co7Ic4K7QuSXBYHtqzFqNw.

The final assembly has a total length of 428.8 Mb in 180 sequence scaffolds with a scaffold N50 of 24.2 Mb ( Table 1). The snail plot in Figure 2 provides a summary of the assembly statistics, while the distribution of assembly scaffolds on GC proportion and coverage is shown in Figure 3. The cumulative assembly plot in Figure 4 shows curves for subsets of scaffolds assigned to different phyla. Most (98.92%) of the assembly sequence was assigned to 19 chromosomal-level scaffolds, representing 18 autosomes and the Z sex chromosome. Chromosome-scale scaffolds confirmed by the Hi-C data are named in order of size ( Figure 5; Table 2). The Z chromosome was identified based on synteny with *Yponomeuta sedellus* (GCA_934045075.1) ( Boyes *et al.*, 2023). While not fully phased, the assembly deposited is of one haplotype. Contigs corresponding to the second haplotype have also been deposited. The mitochondrial genome was also assembled and can be found as a contig within the multifasta file of the genome submission.

**Table 1.** Genome data for *Zelleria hepariella*, ilZelHepa1.1.

Project accession data		
Assembly identifier	ilZelHepa1.1	
Species	*Zelleria hepariella*	
Specimen	ilZelHepa1	
NCBI taxonomy ID	1594360	
BioProject	PRJEB59962	
BioSample ID	SAMEA7746627	
Isolate information	ilZelHepa1: whole organism (DNA sequencing) ilZelHepa2: whole organism (Hi-C sequencing)	
Assembly metrics *		*Benchmark*
Consensus quality (QV)	59.8	*≥ 50*
*k*-mer completeness	100.0%	*≥ 95%*
BUSCO**	C:96.5%[S:95.9%,D:0.6%], F:0.9%,M:2.6%,n:5,286	*C ≥ 95%*
Percentage of assembly mapped to chromosomes	98.92%	*≥ 95%*
Sex chromosomes	Z	*localised homologous pairs*
Organelles	Mitochondrial genome: 16.31 kb	*complete single alleles*
Raw data accessions		
PacificBiosciences SEQUEL II	ERR10906100	
Hi-C Illumina	ERR10908638	
Genome assembly		
Assembly accession	GCA_949319315.1	
*Accession of alternate haplotype*	GCA_949319205.1	
Span (Mb)	428.8	
Number of contigs	896	
Contig N50 length (Mb)	1.0	
Number of scaffolds	180	
Scaffold N50 length (Mb)	24.2	
Longest scaffold (Mb)	40.24	
Genome annotation		
Number of protein-coding genes	15,718	
Number of gene transcripts	15,965	

* Assembly metric benchmarks are adapted from column VGP-2020 of “Table 1: Proposed standards and metrics for defining genome assembly quality” from Rhie *et al.* (2021). ** BUSCO scores based on the lepidoptera_odb10 BUSCO set using version 5.3.2. C = complete [S = single copy, D = duplicated], F = fragmented, M = missing, n = number of orthologues in comparison. A full set of BUSCO scores is available at https://blobtoolkit.genomehubs.org/view/ilZelHepa1_1/dataset/ilZelHepa1_1/busco.

**Table 2.** Chromosomal pseudomolecules in the genome assembly of *Zelleria hepariella*, ilZelHepa1.

INSDC accession	Chromosome	Length (Mb)	GC%
OX439378.1	1	40.24	39.5
OX439380.1	2	33.39	39.5
OX439381.1	3	33.16	39.5
OX439382.1	4	30.6	39.5
OX439383.1	5	26.57	40.0
OX439384.1	6	24.23	40.0
OX439385.1	7	22.64	39.5
OX439386.1	8	19.79	40.5
OX439387.1	9	17.78	39.5
OX439388.1	10	17.06	39.5
OX439389.1	11	16.83	39.5
OX439390.1	12	16.74	39.5
OX439391.1	13	16.56	40.0
OX439392.1	14	16.13	39.5
OX439393.1	15	16.1	39.5
OX439394.1	16	15.29	40.0
OX439395.1	17	14.82	39.5
OX439396.1	18	12.8	40.0
OX439379.1	Z	33.47	40.0
OX439397.1	MT	0.02	17.5

The estimated Quality Value (QV) of the final assembly is 59.8 with *k*-mer completeness of 100.0%, and the assembly has a BUSCO v5.3.2 completeness of 96.5% (single = 95.9%, duplicated = 0.6%), using the lepidoptera_odb10 reference set ( *n* = 5,286).

Metadata for specimens, barcode results, spectra estimates, sequencing runs, contaminants and pre-curation assembly statistics are given at https://links.tol.sanger.ac.uk/species/1594360.

## Genome annotation report

The *Zelleria hepariella* genome assembly (GCA_949319315.1) was annotated at the European Bioinformatics Institute (EBI) on Ensembl Rapid Release. The resulting annotation includes 15,965 transcribed mRNAs from 15,718 protein-coding genes ( Table 1; https://rapid.ensembl.org/Zelleria_hepariella_GCA_949319315.1/Info/Index).

## Methods

### Sample acquisition and nucleic acid extraction

A specimen of *Zelleria hepariella* (specimen ID Ox000820, ToLID ilZelHepa1) was collected from Wytham Woods, Oxfordshire (biological vice-county Berkshire), UK (latitude 51.77, longitude –1.34) on 2020-08-01. The specimen used for Hi-C sequencing (specimen ID Ox001820, ToLID ilZelHepa2) was collected from the same location on 2021-07-24. The specimens were collected in light traps and formally identified by Douglas Boyes (University of Oxford) and then preserved on dry ice.

The workflow for high molecular weight (HMW) DNA extraction at the Wellcome Sanger Institute (WSI) includes a sequence of core procedures: sample preparation; sample homogenisation, DNA extraction, fragmentation, and clean-up. In sample preparation, the ilZelHepa1 sample was weighed and dissected on dry ice ( Jay *et al.*, 2023). The whole organism was homogenised using a PowerMasher II tissue disruptor ( Denton *et al.*, 2023a). HMW DNA was extracted using the Automated MagAttract v1 protocol ( Sheerin *et al.*, 2023). DNA was sheared into an average fragment size of 12–20 kb in a Megaruptor 3 system with speed setting 30 ( Todorovic *et al.*, 2023). Sheared DNA was purified by solid-phase reversible immobilisation ( Strickland *et al.*, 2023): in brief, the method employs a 1.8X ratio of AMPure PB beads to sample to eliminate shorter fragments and concentrate the DNA. The concentration of the sheared and purified DNA was assessed using a Nanodrop spectrophotometer and Qubit Fluorometer and Qubit dsDNA High Sensitivity Assay kit. Fragment size distribution was evaluated by running the sample on the FemtoPulse system.

Protocols developed by the WSI Tree of Life laboratory are publicly available on protocols.io ( Denton *et al.*, 2023b).

### Sequencing

Pacific Biosciences HiFi circular consensus DNA sequencing libraries were constructed according to the manufacturers’ instructions. DNA sequencing was performed by the Scientific Operations core at the WSI on a Pacific Biosciences SEQUEL II (HiFi) instrument. Hi-C data were also generated from the whole organism tissue of ilZelHepa2 using the Arima2 kit and sequenced on the Illumina NovaSeq 6000 instrument.

### Genome assembly, curation and evaluation

Assembly was carried out with Hifiasm ( Cheng *et al.*, 2021) and haplotypic duplication was identified and removed with purge_dups ( Guan *et al.*, 2020). The assembly was then scaffolded with Hi-C data ( Rao *et al.*, 2014) using YaHS ( Zhou *et al.*, 2023). The assembly was checked for contamination and corrected as described previously ( Howe *et al.*, 2021). Manual curation was performed using HiGlass ( Kerpedjiev *et al.*, 2018) and PretextView ( Harry, 2022). The mitochondrial genome was assembled using MitoHiFi ( Uliano-Silva *et al.*, 2023), which runs MitoFinder ( Allio *et al.*, 2020) or MITOS ( Bernt *et al.*, 2013) and uses these annotations to select the final mitochondrial contig and to ensure the general quality of the sequence.

A Hi-C map for the final assembly was produced using bwa-mem2 ( Vasimuddin *et al.*, 2019) in the Cooler file format ( Abdennur & Mirny, 2020). To assess the assembly metrics, the *k*-mer completeness and QV consensus quality values were calculated in Merqury ( Rhie *et al.*, 2020). This work was done using Nextflow ( Di Tommaso *et al.*, 2017) DSL2 pipelines “sanger-tol/readmapping” ( Surana *et al.*, 2023a) and “sanger-tol/genomenote” ( Surana *et al.*, 2023b). The genome was analysed within the BlobToolKit environment ( Challis *et al.*, 2020) and BUSCO scores ( Manni *et al.*, 2021; Simão *et al.*, 2015) were calculated.

Table 3 contains a list of relevant software tool versions and sources.

**Table 3.** Software tools: versions and sources.

Software tool	Version	Source
BlobToolKit	4.2.1	https://github.com/blobtoolkit/blobtoolkit
BUSCO	5.3.2	https://gitlab.com/ezlab/busco
Hifiasm	0.16.1-r375	https://github.com/chhylp123/hifiasm
HiGlass	1.11.6	https://github.com/higlass/higlass
Merqury	MerquryFK	https://github.com/thegenemyers/MERQURY.FK
MitoHiFi	2	https://github.com/marcelauliano/MitoHiFi
PretextView	0.2	https://github.com/wtsi-hpag/PretextView
purge_dups	1.2.3	https://github.com/dfguan/purge_dups
sanger-tol/genomenote	v1.0	https://github.com/sanger-tol/genomenote
sanger-tol/readmapping	1.1.0	https://github.com/sanger-tol/readmapping/tree/1.1.0
YaHS	1.2a	https://github.com/c-zhou/yahs

### Genome annotation

The BRAKER2 pipeline (Brůna *et al.*, 2021) was used in the default protein mode to generate annotation for the *Zelleria hepariella* assembly (GCA_949319315.1) in Ensembl Rapid Release at the EBI.

### Wellcome Sanger Institute – legal and governance

The materials that have contributed to this genome note have been supplied by a Darwin Tree of Life Partner. The submission of materials by a Darwin Tree of Life Partner is subject to the **‘Darwin Tree of Life Project Sampling Code of Practice’**, which can be found in full on the Darwin Tree of Life website here. By agreeing with and signing up to the Sampling Code of Practice, the Darwin Tree of Life Partner agrees they will meet the legal and ethical requirements and standards set out within this document in respect of all samples acquired for, and supplied to, the Darwin Tree of Life Project.

Further, the Wellcome Sanger Institute employs a process whereby due diligence is carried out proportionate to the nature of the materials themselves, and the circumstances under which they have been/are to be collected and provided for use. The purpose of this is to address and mitigate any potential legal and/or ethical implications of receipt and use of the materials as part of the research project, and to ensure that in doing so we align with best practice wherever possible. The overarching areas of consideration are:

•       Ethical review of provenance and sourcing of the material

•       Legality of collection, transfer and use (national and international)

Each transfer of samples is further undertaken according to a Research Collaboration Agreement or Material Transfer Agreement entered into by the Darwin Tree of Life Partner, Genome Research Limited (operating as the Wellcome Sanger Institute), and in some circumstances other Darwin Tree of Life collaborators.

## Data Availability

European Nucleotide Archive: *Zelleria hepariella* (brown ash ermine). Accession number PRJEB59962; https://identifiers.org/ena.embl/PRJEB59962 ( Wellcome Sanger Institute, 2023). The genome sequence is released openly for reuse. The *Zelleria hepariella* genome sequencing initiative is part of the Darwin Tree of Life (DToL) project. All raw sequence data and the assembly have been deposited in INSDC databases. Raw data and assembly accession identifiers are reported in Table 1.

## Author information

Members of the University of Oxford and Wytham Woods Genome Acquisition Lab are listed here: https://doi.org/10.5281/zenodo.7125292.

Members of the Darwin Tree of Life Barcoding collective are listed here: https://doi.org/10.5281/zenodo.4893703.

Members of the Wellcome Sanger Institute Tree of Life Management, Samples and Laboratory team are listed here: https://doi.org/10.5281/zenodo.10066175.

Members of Wellcome Sanger Institute Scientific Operations: Sequencing Operations are listed here: https://doi.org/10.5281/zenodo.10043364.

Members of the Wellcome Sanger Institute Tree of Life Core Informatics team are listed here: https://doi.org/10.5281/zenodo.10066637.

Members of the Tree of Life Core Informatics collective are listed here: https://doi.org/10.5281/zenodo.5013541.

Members of the Darwin Tree of Life Consortium are listed here: https://doi.org/10.5281/zenodo.4783558.
