# Supplementary material for: The genome sequence of the Brown Ash Ermine moth, Zelleria hepariella Stainton, 1849
Source: Wellcome Open Res. 2024 May 9;9:242. [Version 1] doi: 10.12688/wellcomeopenres.21495.1 (PMC11301138; doi:10.12688/wellcomeopenres.21495.1)
Supplement: Peer review history

# Reviewer report: Reviewer response for version 1

**DOI:** 10.21956/wellcomeopenres.23765.r89628

**Published:** 2024-08-05

**Authors:** Ricardo Rodriguez-de-la-vega

**Affiliations:** 1 Écologie Systématique et Évolution, CNRS, Université Paris-Saclay, Paris, France

Comments on Boyes et al., "The genome sequence of the Brown Ash Ermine..."

 The genome assembly presented here attained similar high-quality scores as the other six or so Yponomeutidae already available in Darwin's Tree of Life (DToL) gateway. As always with DToL reports, one misses a relational table about the whole contigs-to-scaffolds-to-chromosomes pipeline. I have no further comments except to point out that the sex of the specimens is stated as "not reported" on the species card at this https://links.tol.sanger.ac.uk/species/1594360, but in the text here is stated as "males".

Are sufficient details of methods and materials provided to allow replication by others?

Yes

Is the rationale for creating the dataset(s) clearly described?

Yes

Are the datasets clearly presented in a useable and accessible format?

Yes

Are the protocols appropriate and is the work technically sound?

Yes

Reviewer Expertise:

Evolutionary genomics, bioinformatics

I confirm that I have read this submission and believe that I have an appropriate level of expertise to confirm that it is of an acceptable scientific standard.

# Reviewer report: Reviewer response for version 1

**DOI:** 10.21956/wellcomeopenres.23765.r88500

**Published:** 2024-07-30

**Authors:** Jing Zhang

**Affiliations:** 1 University of Texas Southwestern Medical Center, Texas, USA

The authors from the Darwin Tree of Life Consortium repeated a protocol similar to their previous work for chromosome-level assemblies, using various sequencing techniques, including short reads, long reads, and Hi-C. The methods were described clearly and in detail, and I have no further questions. My only suggestion for future genome assemblies from the Darwin Tree of Life Consortium is to prioritize female samples so that the W chromosome can also be sequenced.

Are sufficient details of methods and materials provided to allow replication by others?

Yes

Is the rationale for creating the dataset(s) clearly described?

Yes

Are the datasets clearly presented in a useable and accessible format?

Yes

Are the protocols appropriate and is the work technically sound?

Yes

Reviewer Expertise:

Lepidoptera genomics, Computational biology

I confirm that I have read this submission and believe that I have an appropriate level of expertise to confirm that it is of an acceptable scientific standard.
